# 1-{1-[2,8-Bis(trifluoro­meth­yl)-4-quin­olyl]-5-methyl-1*H*-1,2,3-triazol-4-yl}ethanone

**DOI:** 10.1107/S1600536810034926

**Published:** 2010-09-08

**Authors:** H. C. Devarajegowda, S. Jeyaseelan, V. Sumangala, Bojapoojary, Suresh P. Nayak

**Affiliations:** a Department of Physics, Yuvaraja’s College (Constituent College), University of Mysore, Mysore 570 005, Karnataka, India; b Sequent Scientific India Limited, Baikampadi Mangalore, Karnataka, India; c Department of Chemistry, Mangalore University, Mangalagangotri 574 199, Karnataka, India

## Abstract

There are two independent mol­ecules in the asymmetric unit of the title compound, C_16_H_10_F_6_N_4_O. The triazole ring is not coplanar with the quinoline ring system; the dihedral angle between the two planes being 74.47 (12) and 63.97 (13)° in the two mol­ecules. The crystal structure is characterized by inter­molecular C—H⋯F, C—H⋯N and C—H⋯O hydrogen bonding. Weak intra­molecular C—H⋯F inter­actions are observed. Disorder is observed in two F atoms of one of the trifluoro­methyl groups of one independent mol­ecule [occupancy ratios 0.77 (3):0.23 (3) and 0.77 (4):0.23 (4)] and in all three F atoms of one of the trifluoro­methyl groups of the second independent mol­ecule [occupancy ratios 0.520 (14):0.480 (14), 0.615 (17):0.385 (17) and 0.783 (11):0.217 (11)]. The O atom is also disordered over two positions with occupancies of  0.60 (13) and 0.40 (13) in the first mol­ecule.

## Related literature

For general background to triazoles and their benzo derivatives, see: Sanghvi *et al.* (1990 ▶); Bohm & Karow (1981 ▶); Holla *et al.* (2005 ▶); Biagi *et al.* (2004 ▶); Karimkulov *et al.* (1991 ▶); Sherement *et al.* (2004 ▶); Savini *et al.* (1994 ▶); Banu *et al.* (1999 ▶); Julino & Stevens (1998 ▶); Diana & Nitz (1993 ▶); Manfredini *et al.* (2000 ▶); Rene *et al.* (1986 ▶); Passannanti *et al.* (1998 ▶); Deng *et al.* (2008 ▶); Sector & Bardeleben (1971 ▶); Barnard *et al.* (1993 ▶). For a related structure, see: Al-eryani *et al.* (2010 ▶).
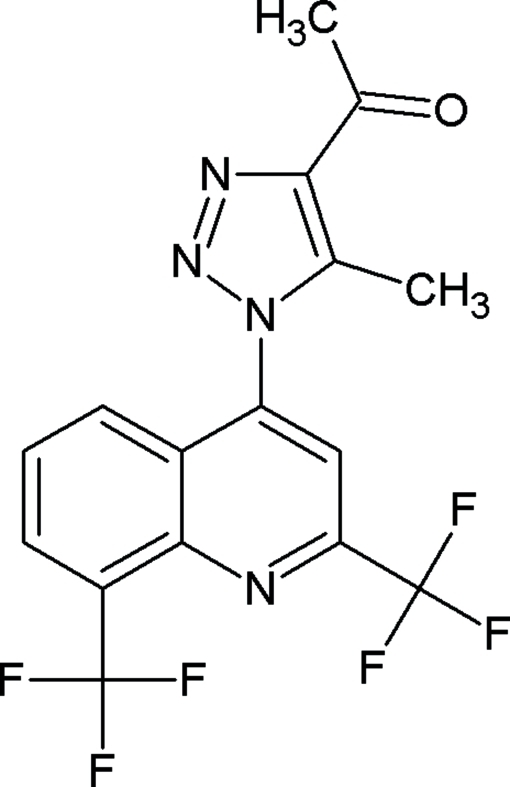


## Experimental

### 

#### Crystal data

C_16_H_10_F_6_N_4_O
*M* *_r_* = 388.28
Monoclinic, 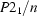

*a* = 14.064 (2) Å
*b* = 8.7275 (13) Å
*c* = 27.468 (4) Å
β = 94.172 (2)°
*V* = 3362.6 (9) Å^3^
*Z* = 8
Mo *K*α radiation
μ = 0.15 mm^−1^
*T* = 293 K
0.20 × 0.20 × 0.15 mm

#### Data collection

Bruker SMART CCD area-detector diffractometer
Absorption correction: ψ scan (*SADABS*; Bruker, 2001 ▶) *T*_min_ = 0.972, *T*_max_ = 0.979
23473 measured reflections
5923 independent reflections
4754 reflections with *I* > 2σ(*I*)
*R*_int_ = 0.028

#### Refinement

*R*[*F* ^2^ > 2σ(*F* ^2^)] = 0.050
*wR*(*F* ^2^) = 0.139
*S* = 1.05
5923 reflections
547 parameters
63 restraints
H-atom parameters constrained
Δρ_max_ = 0.26 e Å^−3^
Δρ_min_ = −0.23 e Å^−3^

### 

Data collection: *SMART* (Bruker, 2001 ▶); cell refinement: *SAINT* (Bruker, 2001 ▶); data reduction: *SAINT*; program(s) used to solve structure: *SHELXS97* (Sheldrick, 2008 ▶); program(s) used to refine structure: *SHELXL97* (Sheldrick, 2008 ▶); molecular graphics: *ORTEP-3* (Farrugia, 1997 ▶); software used to prepare material for publication: *SHELXL97* and *PLATON* (Spek, 2009 ▶).

## Supplementary Material

Crystal structure: contains datablocks I, global. DOI: 10.1107/S1600536810034926/ds2049sup1.cif

Structure factors: contains datablocks I. DOI: 10.1107/S1600536810034926/ds2049Isup2.hkl

Additional supplementary materials:  crystallographic information; 3D view; checkCIF report

## Figures and Tables

**Table 1:** Hydrogen-bond geometry (Å, °)

*D*—H⋯*A*	*D*—H	H⋯*A*	*D*⋯*A*	*D*—H⋯*A*
C5—H5⋯F12	0.93	2.51	3.178 (3)	129
C9—H9⋯N7^i^	0.93	2.47	3.312 (4)	151
C16—H16*A*⋯O2^ii^	0.96	2.49	3.359 (4)	150
C32—H32*B*⋯F5*A*^iii^	0.96	2.41	3.324 (14)	158

Symmetry codes: (i) 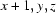; (ii) 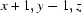; (iii) 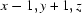.

**Fig. 1.**
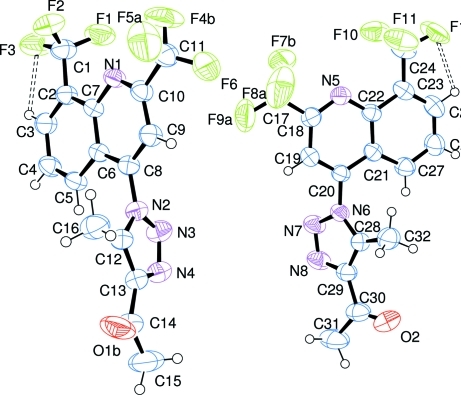
The molecular structure of the title compound. Displacement ellipsoids are drawn at the 50% probability level. Hydrogen atoms are shown as spheres of arbitrary radius and showing intramolecular hydrogen bonds as dashed lines.

**Fig. 2.**
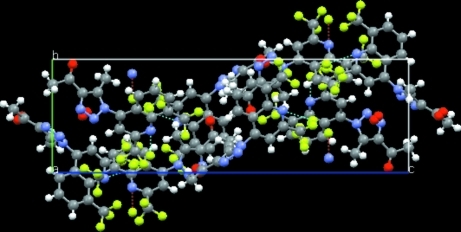
Packing of the molecules showing hydrogen bonds as dashed lines.

## supplementary crystallographic information

### Comment

1,2,3-Triazoles and their benzoderivatives have attracted considerable attention because of their theoretical interest and synthetic value. They also find numerous applications in industry and agriculture due to their extensive biological activitiesand successful application as fluorescent whiteners, light stabilizers and optical brightening agents (Sanghvi *et al.*, 1990).Functionalized 1, 2,3-triazoles constitute one of the common fragments present in biologically active compounds (Bohm *et al.*,1981).This has resulted in a wealth of synthetic methodology for their preparation and incorporation in more complex structures.The accentuated interest in these compounds continues to be expressed in the pharmaceutical community and biological properties of these agents have been the subject of ongoing investigations (Holla *et al.*, 2005). The triazole scaffold has a wide range of therapeutic uses as it is ubiquitously found in drugs. The derivatives of 1, 2, 3-triazoles constitute an important family of heterocyclic compounds due to their chemotherapeutical values (Sanghvi *et al.*, 1990). Some 1,2,3-triazoles are used as DNA cleaving agents and potassium channels activators (Biagi *et al.*, 2004). Since many of them have remarkable antimicrobial(Karimkulov *et al.*, 1991; Sherement *et al.*, 2004), analgesic & anti-inflammatory (Savini *et al.* ,1994), local anesthetic (Banu *et al.* , 1999), antimalarial (Julino *et al.* , 1998), antiviral (Diana *et al.* ,1993), anti-proliferatine (Manfredini *et al.* ,2000), anticonvulsant (Rene *et al.* ,1986), antineoplastic(Passannanti *et al.* , 1998) and anticancer activity (Deng *et al.* ,2008), their synthesis and transformations have been received particular interest for a long time.

Fluorine incorporated compounds exhibit dramatically improved potency compared to their non-fluorinated analogues (Sector *et al.* ,1971) since its incorporation alters the electronic, lipophilic and steric parameters and can critically increase the intrinsic activity,chemical and metabolic stability.In particular, introduction of CF_3_ group in organic molecules immensely increased the pharmacological activity as well as lipophilicity (Barnard *et al.* ,1993).

The structure of 1-{1-[2,8-bis(trifluoromethyl) quinolin-4-*yl*]-5- methyl-1*H*-1,2,3-triazol-4-*yl*}ethanone contains two independent molecules in the asymmetric unit. The triazole ring is not coplanar with the quinoline ring system; the dihedral angle between the two planes is 74.21 (08)°. The structure of the molecules is stabilized by intermolecular C5–H5···F12 & C9–H9···N7, C16–H16A···O2 & C32–H32B···F5A and weak intramolecular C3–H3···F3 & C25–H25···F12 hydrogen bonding (Table 1) and shows two fluorine atoms disordered in one of the trifluoromethyl group and oxygen atom.

### Experimental

A solution of 4-Azido-2,8 bis trifluoro methyl quinoline (2.5 g,0.08 mol) in 25 mL me thanol was treated with acetyl acetone (0.9 g, 0.008 mol) and the mixture was cooled to 0°C. Sodium methoxide (0.008 mol) was added in lots under nitrogen over a period of 30 minutes. It was then stirred for 30 minutes. The progress of the reaction was monitored by TLC using ethyl acetate: hexane (1: 4, *v*/*v*) as mobile phase. The reaction mass is quenched to ice water. The title compound is isolated by filtration as a yellow solid. The recrystallization of the compound in methanol gave 76% of pure compound. Melting point: 427 K. Mol.Wt: 388.27 IR (KBr, γ/cm^-1^): 3025 (Ar—H), 1715 (C=O), 1005(C—F). ^1^H NMR (300 MHz, CDCl_3_): δ, 2.52(s, CH_3_, 3H), 2.83(s, 3H, COCH_3_), 7.26(s, Ar—H,1*H*), 7.65–7.68(d, 1H, Ar—H, J =8.4 Hz),8.32–8.34 (d, 1H, Ar—H, J =7.2 Hz), 7.83–7. 86 (t, IH, Ar—H, J =7.8 Hz). MS (m/*z*, %):388 (*M*^+^). Anal. calcd for C_16_ H_10_F_6_N_4_O (in %): C-49.47, H-2.61, N-14.44. Found C-49.49, H-2.60, N-14.43.

### Refinement

All H atoms were positioned at calculated positions with C—H = 0.93Å for aromatic H and 0.96Å for methyl H and refined using a riding model with *U*_iso_(H) =1.5*U*_eq_(C)for methyl H and 1.2*U*_eq_(C) for other and also refined two fluorine atoms of the trifluoromethyl group is disordered with two orientations.

variation in *U*_eq_ for C1, C11, C17 and C24 as compared to Neighbors

C1, C11, C17 and C24 are free terminal trifluoromethyl carbon attached to benzene ring of quinoline part of the structure. The high electron dense fluorine atoms, freely movable in this case, increase the thermal factor of the C1, C11, C17 and C24. Thus giving variation in *U*_eq_ as Compared to Neighbors

### Crystal data

**Table:**

C_16_H_10_F_6_N_4_O	*F*(000) = 1568
*M**_r_* = 388.28	*D*_x_ = 1.534 Mg m^−^^3^
Monoclinic, *P*2_1_/*n*	Melting point: 427 K
Hall symbol: -P 2yn	Mo *K*α radiation, λ = 0.71073 Å
*a* = 14.064 (2) Å	Cell parameters from 5923 reflections
*b* = 8.7275 (13) Å	θ = 1.5–25.0°
*c* = 27.468 (4) Å	µ = 0.14 mm^−^^1^
β = 94.172 (2)°	*T* = 293 K
*V* = 3362.6 (9) Å^3^	Plate, colourless
*Z* = 8	0.20 × 0.20 × 0.15 mm

### Data collection

**Table:**

Bruker SMART CCD area-detector diffractometer	5923 independent reflections
Radiation source: fine-focus sealed tube	4754 reflections with *I* > 2σ(*I*)
graphite	*R*_int_ = 0.028
ω and φ scans	θ_max_ = 25.0°, θ_min_ = 1.5°
Absorption correction: ψ scan (*SADABS*; Bruker, 2001)	*h* = −16→16
*T*_min_ = 0.972, *T*_max_ = 0.979	*k* = −10→10
23473 measured reflections	*l* = −32→30

### Refinement

**Table:**

Refinement on *F*^2^	Primary atom site location: structure-invariant direct methods
Least-squares matrix: full	Secondary atom site location: difference Fourier map
*R*[*F*^2^ > 2σ(*F*^2^)] = 0.050	Hydrogen site location: inferred from neighbouring sites
*wR*(*F*^2^) = 0.139	H-atom parameters constrained
*S* = 1.05	*w* = 1/[σ^2^(*F*_o_^2^) + (0.069*P*)^2^ + 1.1626*P*] where *P* = (*F*_o_^2^ + 2*F*_c_^2^)/3
5923 reflections	(Δ/σ)_max_ < 0.001
547 parameters	Δρ_max_ = 0.26 e Å^−^^3^
63 restraints	Δρ_min_ = −0.23 e Å^−^^3^

### Special details

**Table:**

Geometry. All s.u.'s (except the s.u. in the dihedral angle between two l.s. planes) are estimated using the full covariance matrix. The cell s.u.'s are taken into account individually in the estimation of s.u.'s in distances, angles and torsion angles; correlations between s.u.'s in cell parameters are only used when they are defined by crystal symmetry. An approximate (isotropic) treatment of cell s.u.'s is used for estimating s.u.'s involving l.s. planes.
Refinement. Refinement of *F*^2^ against ALL reflections. The weighted *R*-factor *wR* and goodness of fit *S* are based on *F*^2^, conventional *R*-factors *R* are based on *F*, with *F* set to zero for negative *F*^2^. The threshold expression of *F*^2^ > 2σ(*F*^2^) is used only for calculating *R*-factors(gt) *etc*. and is not relevant to the choice of reflections for refinement. *R*-factors based on *F*^2^ are statistically about twice as large as those based on *F*, and *R*- factors based on ALL data will be even larger.

### Fractional atomic coordinates and isotropic or equivalent isotropic displacement parameters (Å^2^)

**Table:**

	*x*	*y*	*z*	*U*_iso_*/*U*_eq_	Occ. (<1)
F1	0.62603 (13)	−0.2838 (2)	0.18151 (6)	0.0844 (5)	
F2	0.75645 (12)	−0.3765 (2)	0.16020 (6)	0.0801 (5)	
F3	0.62542 (16)	−0.4783 (2)	0.13491 (7)	0.1023 (7)	
F4B	0.8238 (11)	0.0406 (9)	0.23119 (18)	0.128 (3)	0.77 (3)
F4A	0.7910 (14)	0.083 (3)	0.2312 (5)	0.078 (5)	0.23 (3)
F5B	0.9284 (8)	0.158 (3)	0.1861 (9)	0.094 (6)	0.23 (4)
F5A	0.9304 (3)	0.101 (2)	0.1948 (5)	0.135 (3)	0.77 (4)
F6	0.82879 (16)	0.2748 (2)	0.20693 (7)	0.0989 (6)	
F7B	0.2388 (9)	0.1504 (13)	0.27486 (16)	0.134 (5)	0.520 (14)
F7A	0.1679 (8)	0.0748 (7)	0.2564 (4)	0.121 (4)	0.480 (14)
F8B	0.2743 (9)	0.2284 (13)	0.2066 (6)	0.135 (6)	0.385 (17)
F8A	0.2871 (4)	0.2331 (8)	0.2421 (6)	0.150 (4)	0.615 (17)
F9B	0.1555 (9)	0.0653 (10)	0.2269 (7)	0.096 (6)	0.217 (11)
F9A	0.1964 (4)	0.1177 (6)	0.19132 (18)	0.128 (2)	0.783 (11)
F10	0.06252 (14)	0.3249 (2)	0.37351 (6)	0.0909 (6)	
F11	0.17217 (12)	0.4920 (3)	0.37357 (6)	0.0939 (6)	
F12	0.04672 (15)	0.5362 (3)	0.41025 (6)	0.1034 (7)	
O1B	0.8131 (8)	0.499 (5)	−0.0850 (17)	0.094 (6)	0.40 (13)
O1A	0.8059 (15)	0.466 (5)	−0.0966 (16)	0.088 (6)	0.60 (13)
O2	−0.05430 (19)	0.9546 (3)	0.05710 (9)	0.0934 (7)	
N1	0.75116 (13)	−0.0489 (2)	0.14556 (6)	0.0459 (4)	
N2	0.70539 (13)	0.2637 (2)	0.02946 (7)	0.0448 (4)	
N3	0.61525 (14)	0.3219 (2)	0.02063 (8)	0.0568 (5)	
N4	0.61674 (15)	0.4042 (2)	−0.01854 (8)	0.0576 (5)	
N5	0.11417 (13)	0.3718 (2)	0.27600 (7)	0.0504 (5)	
N6	−0.00853 (13)	0.5686 (2)	0.14652 (6)	0.0466 (4)	
N7	−0.06553 (16)	0.4771 (2)	0.11648 (7)	0.0602 (6)	
N8	−0.09389 (16)	0.5602 (3)	0.07907 (8)	0.0648 (6)	
C1	0.6679 (2)	−0.3437 (3)	0.14425 (10)	0.0590 (7)	
C2	0.66029 (16)	−0.2409 (3)	0.10050 (8)	0.0456 (5)	
C3	0.61058 (17)	−0.2867 (3)	0.05863 (9)	0.0518 (6)	
H3	0.5842	−0.3844	0.0568	0.062*	
C4	0.59872 (17)	−0.1888 (3)	0.01827 (8)	0.0527 (6)	
H4	0.5662	−0.2234	−0.0103	0.063*	
C5	0.63381 (16)	−0.0448 (3)	0.02028 (8)	0.0469 (5)	
H5	0.6243	0.0197	−0.0066	0.056*	
C6	0.68504 (14)	0.0080 (2)	0.06324 (7)	0.0405 (5)	
C7	0.70045 (14)	−0.0920 (2)	0.10371 (7)	0.0401 (5)	
C8	0.72286 (15)	0.1571 (2)	0.06873 (8)	0.0422 (5)	
C9	0.77317 (16)	0.1999 (3)	0.11060 (8)	0.0493 (5)	
H9	0.7988	0.2978	0.1144	0.059*	
C10	0.78488 (16)	0.0901 (3)	0.14781 (8)	0.0480 (5)	
C11	0.8398 (2)	0.1312 (3)	0.19515 (10)	0.0672 (8)	
C12	0.76358 (16)	0.3096 (3)	−0.00439 (8)	0.0488 (5)	
C13	0.70503 (18)	0.4002 (3)	−0.03525 (9)	0.0523 (6)	
C14	0.7286 (3)	0.4792 (3)	−0.07976 (10)	0.0699 (8)	
C15	0.6496 (3)	0.5594 (5)	−0.10873 (13)	0.1034 (12)	
H15A	0.6740	0.6064	−0.1369	0.155*	
H15B	0.6230	0.6367	−0.0889	0.155*	
H15C	0.6010	0.4867	−0.1191	0.155*	
C16	0.86421 (19)	0.2661 (4)	−0.00449 (11)	0.0729 (8)	
H16A	0.8809	0.2009	0.0230	0.109*	
H16B	0.9031	0.3566	−0.0023	0.109*	
H16C	0.8745	0.2124	−0.0341	0.109*	
C17	0.2014 (2)	0.1926 (3)	0.23226 (11)	0.0745 (8)	
C18	0.13305 (17)	0.3238 (3)	0.23277 (9)	0.0523 (6)	
C19	0.09522 (17)	0.3874 (3)	0.18863 (9)	0.0514 (6)	
H19	0.1123	0.3498	0.1588	0.062*	
C20	0.03335 (15)	0.5047 (2)	0.19089 (8)	0.0431 (5)	
C21	0.00741 (15)	0.5608 (3)	0.23647 (8)	0.0443 (5)	
C22	0.05239 (15)	0.4897 (3)	0.27825 (8)	0.0442 (5)	
C23	0.03100 (17)	0.5448 (3)	0.32524 (8)	0.0526 (6)	
C24	0.0786 (2)	0.4748 (4)	0.36993 (10)	0.0670 (7)	
C25	−0.03136 (19)	0.6618 (3)	0.32868 (10)	0.0636 (7)	
H25	−0.0440	0.6983	0.3593	0.076*	
C26	−0.07694 (19)	0.7285 (3)	0.28722 (10)	0.0645 (7)	
H26	−0.1201	0.8078	0.2906	0.077*	
C27	−0.05890 (17)	0.6790 (3)	0.24192 (9)	0.0537 (6)	
H27	−0.0904	0.7233	0.2145	0.064*	
C28	−0.00056 (16)	0.7101 (3)	0.12737 (8)	0.0483 (5)	
C29	−0.05686 (17)	0.7031 (3)	0.08470 (8)	0.0521 (6)	
C30	−0.07782 (19)	0.8242 (4)	0.04788 (10)	0.0658 (7)	
C31	−0.1272 (2)	0.7785 (5)	0.00060 (11)	0.0913 (11)	
H31A	−0.1367	0.8671	−0.0199	0.137*	
H31B	−0.1879	0.7340	0.0062	0.137*	
H31C	−0.0891	0.7049	−0.0152	0.137*	
C32	0.0637 (2)	0.8291 (3)	0.14926 (11)	0.0700 (8)	
H32A	0.0917	0.7937	0.1801	0.105*	
H32B	0.0280	0.9210	0.1540	0.105*	
H32C	0.1131	0.8500	0.1279	0.105*	

### Atomic displacement parameters (Å^2^)

**Table:**

	*U*^11^	*U*^22^	*U*^33^	*U*^12^	*U*^13^	*U*^23^
F1	0.0980 (12)	0.1025 (13)	0.0544 (10)	0.0095 (10)	0.0171 (9)	0.0280 (9)
F2	0.0782 (11)	0.0789 (11)	0.0806 (11)	0.0107 (8)	−0.0127 (9)	0.0266 (9)
F3	0.1420 (17)	0.0650 (11)	0.0946 (13)	−0.0417 (11)	−0.0284 (12)	0.0313 (10)
F4B	0.233 (7)	0.082 (3)	0.058 (2)	−0.045 (4)	−0.070 (3)	0.0165 (17)
F4A	0.094 (8)	0.112 (9)	0.028 (5)	−0.024 (6)	0.007 (5)	0.003 (5)
F5B	0.073 (8)	0.099 (9)	0.101 (8)	−0.024 (8)	−0.042 (6)	0.003 (7)
F5A	0.086 (3)	0.183 (8)	0.127 (5)	0.044 (3)	−0.058 (2)	−0.064 (5)
F6	0.1453 (18)	0.0743 (12)	0.0709 (11)	−0.0031 (11)	−0.0348 (11)	−0.0232 (9)
F7B	0.169 (8)	0.152 (7)	0.076 (3)	0.113 (7)	−0.026 (4)	0.003 (3)
F7A	0.180 (8)	0.074 (4)	0.110 (6)	0.047 (4)	0.015 (6)	0.025 (4)
F8B	0.132 (8)	0.138 (7)	0.144 (9)	0.073 (6)	0.068 (6)	0.044 (6)
F8A	0.083 (3)	0.130 (4)	0.227 (9)	0.047 (3)	−0.063 (4)	−0.063 (5)
F9B	0.115 (8)	0.050 (5)	0.117 (11)	0.010 (5)	−0.042 (7)	−0.037 (6)
F9A	0.142 (4)	0.115 (3)	0.121 (3)	0.067 (3)	−0.026 (3)	−0.054 (3)
F10	0.1089 (14)	0.0942 (13)	0.0684 (11)	−0.0121 (11)	−0.0012 (10)	0.0312 (10)
F11	0.0689 (11)	0.1451 (17)	0.0648 (11)	−0.0185 (11)	−0.0156 (8)	0.0198 (11)
F12	0.1265 (16)	0.1486 (18)	0.0351 (9)	0.0113 (13)	0.0062 (9)	−0.0014 (10)
O1B	0.129 (13)	0.106 (10)	0.047 (10)	−0.015 (10)	0.021 (5)	0.023 (7)
O1A	0.094 (6)	0.114 (9)	0.059 (9)	0.024 (4)	0.031 (4)	0.029 (7)
O2	0.1241 (19)	0.0700 (14)	0.0847 (15)	0.0196 (13)	−0.0017 (13)	0.0242 (12)
N1	0.0517 (11)	0.0504 (11)	0.0349 (10)	−0.0014 (9)	−0.0021 (8)	0.0016 (8)
N2	0.0466 (10)	0.0431 (10)	0.0442 (10)	−0.0013 (8)	0.0004 (8)	0.0059 (8)
N3	0.0491 (11)	0.0599 (12)	0.0609 (13)	0.0026 (9)	0.0012 (9)	0.0120 (10)
N4	0.0615 (13)	0.0535 (12)	0.0567 (13)	0.0052 (10)	−0.0027 (10)	0.0111 (10)
N5	0.0528 (11)	0.0510 (11)	0.0465 (11)	−0.0011 (9)	−0.0011 (9)	0.0046 (9)
N6	0.0521 (11)	0.0486 (11)	0.0383 (10)	−0.0030 (8)	−0.0022 (8)	−0.0018 (8)
N7	0.0709 (14)	0.0609 (13)	0.0465 (12)	−0.0131 (10)	−0.0121 (10)	0.0015 (10)
N8	0.0671 (14)	0.0775 (16)	0.0476 (12)	−0.0073 (12)	−0.0113 (10)	0.0034 (11)
C1	0.0687 (17)	0.0540 (15)	0.0529 (15)	−0.0091 (12)	−0.0049 (13)	0.0107 (12)
C2	0.0496 (12)	0.0448 (12)	0.0420 (12)	−0.0032 (10)	0.0009 (10)	0.0040 (9)
C3	0.0579 (14)	0.0452 (13)	0.0515 (14)	−0.0080 (11)	−0.0014 (11)	−0.0018 (11)
C4	0.0576 (14)	0.0581 (14)	0.0411 (13)	−0.0067 (11)	−0.0049 (10)	−0.0056 (11)
C5	0.0536 (13)	0.0528 (13)	0.0336 (11)	−0.0031 (11)	−0.0013 (9)	0.0021 (10)
C6	0.0409 (11)	0.0458 (12)	0.0348 (11)	−0.0006 (9)	0.0035 (9)	0.0015 (9)
C7	0.0426 (11)	0.0447 (12)	0.0328 (11)	−0.0013 (9)	0.0008 (9)	−0.0003 (9)
C8	0.0421 (11)	0.0460 (12)	0.0386 (12)	−0.0017 (9)	0.0034 (9)	0.0040 (9)
C9	0.0533 (13)	0.0459 (12)	0.0480 (13)	−0.0092 (10)	−0.0018 (10)	−0.0011 (10)
C10	0.0498 (13)	0.0514 (13)	0.0418 (12)	−0.0037 (10)	−0.0035 (10)	−0.0032 (10)
C11	0.084 (2)	0.0591 (17)	0.0552 (17)	−0.0057 (15)	−0.0206 (15)	−0.0056 (13)
C12	0.0575 (13)	0.0462 (12)	0.0434 (12)	0.0015 (10)	0.0076 (10)	0.0065 (10)
C13	0.0639 (15)	0.0460 (13)	0.0470 (13)	0.0038 (11)	0.0038 (11)	0.0051 (10)
C14	0.093 (2)	0.0618 (17)	0.0549 (17)	0.0063 (16)	0.0078 (16)	0.0152 (13)
C15	0.127 (3)	0.106 (3)	0.077 (2)	0.024 (2)	0.003 (2)	0.044 (2)
C16	0.0649 (16)	0.084 (2)	0.0722 (19)	0.0182 (15)	0.0223 (14)	0.0243 (15)
C17	0.088 (2)	0.0654 (19)	0.070 (2)	0.0224 (17)	−0.0001 (18)	0.0005 (16)
C18	0.0547 (13)	0.0499 (13)	0.0519 (15)	0.0016 (11)	0.0009 (11)	0.0016 (11)
C19	0.0601 (14)	0.0510 (13)	0.0430 (13)	0.0010 (11)	0.0031 (11)	−0.0045 (10)
C20	0.0472 (12)	0.0442 (12)	0.0372 (12)	−0.0039 (9)	−0.0008 (9)	0.0015 (9)
C21	0.0423 (11)	0.0468 (12)	0.0435 (12)	−0.0046 (9)	0.0014 (9)	−0.0021 (10)
C22	0.0438 (12)	0.0480 (13)	0.0404 (12)	−0.0067 (10)	−0.0003 (9)	0.0018 (9)
C23	0.0535 (13)	0.0636 (15)	0.0406 (13)	−0.0104 (12)	0.0033 (10)	−0.0008 (11)
C24	0.0702 (18)	0.086 (2)	0.0445 (15)	−0.0115 (15)	0.0008 (12)	0.0076 (13)
C25	0.0677 (16)	0.0779 (18)	0.0465 (14)	−0.0004 (14)	0.0120 (12)	−0.0108 (13)
C26	0.0644 (16)	0.0702 (17)	0.0599 (17)	0.0123 (13)	0.0110 (13)	−0.0067 (13)
C27	0.0518 (13)	0.0611 (15)	0.0476 (14)	0.0039 (11)	−0.0004 (11)	0.0021 (11)
C28	0.0529 (13)	0.0479 (13)	0.0441 (13)	0.0045 (10)	0.0033 (10)	0.0014 (10)
C29	0.0505 (13)	0.0604 (15)	0.0455 (13)	0.0068 (11)	0.0031 (10)	0.0055 (11)
C30	0.0598 (15)	0.081 (2)	0.0567 (16)	0.0181 (14)	0.0061 (12)	0.0182 (15)
C31	0.086 (2)	0.122 (3)	0.0627 (19)	0.021 (2)	−0.0116 (16)	0.0249 (19)
C32	0.087 (2)	0.0544 (15)	0.0669 (18)	−0.0120 (14)	−0.0050 (15)	0.0025 (13)

### Geometric parameters (Å, °)

**Table:**

F1—C1	1.325 (3)	C5—H5	0.9300
F2—C1	1.321 (3)	C6—C8	1.410 (3)
F3—C1	1.334 (3)	C6—C7	1.418 (3)
F4B—C11	1.300 (4)	C8—C9	1.358 (3)
F4A—C11	1.313 (7)	C9—C10	1.402 (3)
F5B—C11	1.309 (8)	C9—H9	0.9300
F5A—C11	1.301 (5)	C10—C11	1.507 (3)
F6—C11	1.306 (3)	C12—C13	1.386 (3)
F7B—C17	1.301 (5)	C12—C16	1.465 (3)
F7A—C17	1.328 (5)	C13—C14	1.462 (4)
F8B—C17	1.324 (6)	C14—C15	1.493 (4)
F8A—C17	1.267 (5)	C15—H15A	0.9600
F9B—C17	1.288 (7)	C15—H15B	0.9600
F9A—C17	1.298 (4)	C15—H15C	0.9600
F10—C24	1.333 (3)	C16—H16A	0.9600
F11—C24	1.321 (3)	C16—H16B	0.9600
F12—C24	1.337 (3)	C16—H16C	0.9600
O1B—C14	1.220 (9)	C17—C18	1.496 (4)
O1A—C14	1.217 (7)	C18—C19	1.402 (3)
O2—C30	1.207 (4)	C19—C20	1.348 (3)
N1—C10	1.303 (3)	C19—H19	0.9300
N1—C7	1.361 (3)	C20—C21	1.417 (3)
N2—C12	1.344 (3)	C21—C27	1.406 (3)
N2—N3	1.371 (3)	C21—C22	1.413 (3)
N2—C8	1.432 (3)	C22—C23	1.429 (3)
N3—N4	1.295 (3)	C23—C25	1.354 (4)
N4—C13	1.355 (3)	C23—C24	1.486 (4)
N5—C18	1.304 (3)	C25—C26	1.393 (4)
N5—C22	1.352 (3)	C25—H25	0.9300
N6—C28	1.350 (3)	C26—C27	1.358 (3)
N6—N7	1.366 (3)	C26—H26	0.9300
N6—C20	1.428 (3)	C27—H27	0.9300
N7—N8	1.297 (3)	C28—C29	1.367 (3)
N8—C29	1.356 (3)	C28—C32	1.475 (4)
C1—C2	1.497 (3)	C29—C30	1.478 (4)
C2—C3	1.362 (3)	C30—C31	1.482 (4)
C2—C7	1.417 (3)	C31—H31A	0.9600
C3—C4	1.400 (3)	C31—H31B	0.9600
C3—H3	0.9300	C31—H31C	0.9600
C4—C5	1.350 (3)	C32—H32A	0.9600
C4—H4	0.9300	C32—H32B	0.9600
C5—C6	1.414 (3)	C32—H32C	0.9600
			
C10—N1—C7	117.63 (19)	H16A—C16—H16B	109.5
C12—N2—N3	111.66 (18)	C12—C16—H16C	109.5
C12—N2—C8	129.43 (18)	H16A—C16—H16C	109.5
N3—N2—C8	118.67 (17)	H16B—C16—H16C	109.5
N4—N3—N2	106.37 (18)	F8A—C17—F9B	136.6 (7)
N3—N4—C13	109.73 (19)	F8A—C17—F9A	108.5 (6)
C18—N5—C22	117.40 (19)	F9B—C17—F9A	58.2 (8)
C28—N6—N7	111.18 (18)	F8A—C17—F7B	64.5 (8)
C28—N6—C20	130.48 (19)	F9B—C17—F7B	91.4 (8)
N7—N6—C20	118.33 (18)	F9A—C17—F7B	129.0 (4)
N8—N7—N6	106.56 (19)	F8A—C17—F8B	44.4 (5)
N7—N8—C29	109.3 (2)	F9B—C17—F8B	123.0 (11)
F2—C1—F1	106.9 (2)	F9A—C17—F8B	69.7 (9)
F2—C1—F3	105.7 (2)	F7B—C17—F8B	105.5 (9)
F1—C1—F3	106.1 (2)	F8A—C17—F7A	118.4 (8)
F2—C1—C2	114.0 (2)	F9B—C17—F7A	36.5 (8)
F1—C1—C2	111.9 (2)	F9A—C17—F7A	92.6 (6)
F3—C1—C2	111.6 (2)	F7B—C17—F7A	57.8 (5)
C3—C2—C7	120.0 (2)	F8B—C17—F7A	139.9 (5)
C3—C2—C1	120.3 (2)	F8A—C17—C18	112.7 (3)
C7—C2—C1	119.6 (2)	F9B—C17—C18	110.2 (6)
C2—C3—C4	120.9 (2)	F9A—C17—C18	113.5 (3)
C2—C3—H3	119.6	F7B—C17—C18	115.2 (3)
C4—C3—H3	119.6	F8B—C17—C18	110.4 (4)
C5—C4—C3	120.9 (2)	F7A—C17—C18	109.7 (4)
C5—C4—H4	119.5	N5—C18—C19	124.8 (2)
C3—C4—H4	119.5	N5—C18—C17	115.3 (2)
C4—C5—C6	120.0 (2)	C19—C18—C17	119.9 (2)
C4—C5—H5	120.0	C20—C19—C18	117.8 (2)
C6—C5—H5	120.0	C20—C19—H19	121.1
C8—C6—C5	123.73 (19)	C18—C19—H19	121.1
C8—C6—C7	116.70 (18)	C19—C20—C21	120.8 (2)
C5—C6—C7	119.57 (19)	C19—C20—N6	119.0 (2)
N1—C7—C2	119.19 (19)	C21—C20—N6	120.17 (19)
N1—C7—C6	122.24 (19)	C27—C21—C22	119.8 (2)
C2—C7—C6	118.56 (19)	C27—C21—C20	124.3 (2)
C9—C8—C6	120.9 (2)	C22—C21—C20	115.9 (2)
C9—C8—N2	120.6 (2)	N5—C22—C21	123.3 (2)
C6—C8—N2	118.44 (18)	N5—C22—C23	118.4 (2)
C8—C9—C10	117.1 (2)	C21—C22—C23	118.3 (2)
C8—C9—H9	121.4	C25—C23—C22	119.8 (2)
C10—C9—H9	121.4	C25—C23—C24	120.5 (2)
N1—C10—C9	125.4 (2)	C22—C23—C24	119.7 (2)
N1—C10—C11	115.1 (2)	F11—C24—F10	106.2 (3)
C9—C10—C11	119.5 (2)	F11—C24—F12	106.5 (2)
F4B—C11—F5A	96.2 (11)	F10—C24—F12	105.3 (2)
F4B—C11—F6	111.4 (5)	F11—C24—C23	113.7 (2)
F5A—C11—F6	109.1 (8)	F10—C24—C23	113.3 (2)
F4B—C11—F5B	118.2 (13)	F12—C24—C23	111.2 (3)
F5A—C11—F5B	24.2 (9)	C23—C25—C26	121.3 (2)
F6—C11—F5B	90.5 (12)	C23—C25—H25	119.3
F4B—C11—F4A	26.3 (8)	C26—C25—H25	119.3
F5A—C11—F4A	120.5 (13)	C27—C26—C25	120.7 (2)
F6—C11—F4A	92.3 (11)	C27—C26—H26	119.7
F5B—C11—F4A	139.1 (14)	C25—C26—H26	119.7
F4B—C11—C10	114.0 (4)	C26—C27—C21	120.0 (2)
F5A—C11—C10	112.7 (4)	C26—C27—H27	120.0
F6—C11—C10	112.3 (2)	C21—C27—H27	120.0
F5B—C11—C10	108.3 (11)	N6—C28—C29	103.5 (2)
F4A—C11—C10	108.2 (8)	N6—C28—C32	123.4 (2)
N2—C12—C13	103.2 (2)	C29—C28—C32	132.8 (2)
N2—C12—C16	124.1 (2)	N8—C29—C28	109.4 (2)
C13—C12—C16	132.7 (2)	N8—C29—C30	121.6 (2)
N4—C13—C12	109.0 (2)	C28—C29—C30	129.0 (3)
N4—C13—C14	122.7 (2)	O2—C30—C29	119.5 (3)
C12—C13—C14	128.3 (2)	O2—C30—C31	122.8 (3)
O1A—C14—O1B	20.3 (18)	C29—C30—C31	117.7 (3)
O1A—C14—C13	122.5 (9)	C30—C31—H31A	109.5
O1B—C14—C13	116.5 (15)	C30—C31—H31B	109.5
O1A—C14—C15	119.4 (12)	H31A—C31—H31B	109.5
O1B—C14—C15	124.5 (13)	C30—C31—H31C	109.5
C13—C14—C15	117.5 (3)	H31A—C31—H31C	109.5
C14—C15—H15A	109.5	H31B—C31—H31C	109.5
C14—C15—H15B	109.5	C28—C32—H32A	109.5
H15A—C15—H15B	109.5	C28—C32—H32B	109.5
C14—C15—H15C	109.5	H32A—C32—H32B	109.5
H15A—C15—H15C	109.5	C28—C32—H32C	109.5
H15B—C15—H15C	109.5	H32A—C32—H32C	109.5
C12—C16—H16A	109.5	H32B—C32—H32C	109.5
C12—C16—H16B	109.5		
			
C12—N2—N3—N4	−0.1 (3)	C12—C13—C14—O1B	−19 (3)
C8—N2—N3—N4	−174.99 (19)	N4—C13—C14—C15	−3.4 (4)
N2—N3—N4—C13	0.2 (3)	C12—C13—C14—C15	175.0 (3)
C28—N6—N7—N8	−0.6 (3)	C22—N5—C18—C19	1.1 (4)
C20—N6—N7—N8	−179.6 (2)	C22—N5—C18—C17	−179.7 (2)
N6—N7—N8—C29	−0.5 (3)	F8A—C17—C18—N5	−76.4 (9)
F2—C1—C2—C3	122.6 (3)	F9B—C17—C18—N5	96.7 (10)
F1—C1—C2—C3	−115.9 (3)	F9A—C17—C18—N5	159.8 (4)
F3—C1—C2—C3	2.9 (4)	F7B—C17—C18—N5	−4.9 (9)
F2—C1—C2—C7	−61.0 (3)	F8B—C17—C18—N5	−124.3 (10)
F1—C1—C2—C7	60.5 (3)	F7A—C17—C18—N5	57.8 (7)
F3—C1—C2—C7	179.3 (2)	F8A—C17—C18—C19	102.8 (8)
C7—C2—C3—C4	0.3 (4)	F9B—C17—C18—C19	−84.1 (10)
C1—C2—C3—C4	176.7 (2)	F9A—C17—C18—C19	−21.0 (5)
C2—C3—C4—C5	−2.0 (4)	F7B—C17—C18—C19	174.3 (8)
C3—C4—C5—C6	1.3 (4)	F8B—C17—C18—C19	55.0 (11)
C4—C5—C6—C8	−179.0 (2)	F7A—C17—C18—C19	−123.0 (7)
C4—C5—C6—C7	1.0 (3)	N5—C18—C19—C20	−1.4 (4)
C10—N1—C7—C2	−178.2 (2)	C17—C18—C19—C20	179.5 (2)
C10—N1—C7—C6	0.9 (3)	C18—C19—C20—C21	−0.3 (3)
C3—C2—C7—N1	−179.0 (2)	C18—C19—C20—N6	−178.1 (2)
C1—C2—C7—N1	4.6 (3)	C28—N6—C20—C19	−115.8 (3)
C3—C2—C7—C6	1.9 (3)	N7—N6—C20—C19	63.0 (3)
C1—C2—C7—C6	−174.5 (2)	C28—N6—C20—C21	66.3 (3)
C8—C6—C7—N1	−1.6 (3)	N7—N6—C20—C21	−114.9 (2)
C5—C6—C7—N1	178.4 (2)	C19—C20—C21—C27	−177.8 (2)
C8—C6—C7—C2	177.44 (19)	N6—C20—C21—C27	0.0 (3)
C5—C6—C7—C2	−2.6 (3)	C19—C20—C21—C22	1.9 (3)
C5—C6—C8—C9	−178.6 (2)	N6—C20—C21—C22	179.73 (18)
C7—C6—C8—C9	1.4 (3)	C18—N5—C22—C21	0.7 (3)
C5—C6—C8—N2	2.6 (3)	C18—N5—C22—C23	−179.7 (2)
C7—C6—C8—N2	−177.39 (18)	C27—C21—C22—N5	177.5 (2)
C12—N2—C8—C9	79.1 (3)	C20—C21—C22—N5	−2.2 (3)
N3—N2—C8—C9	−107.1 (2)	C27—C21—C22—C23	−2.1 (3)
C12—N2—C8—C6	−102.1 (3)	C20—C21—C22—C23	178.20 (19)
N3—N2—C8—C6	71.7 (3)	N5—C22—C23—C25	−179.3 (2)
C6—C8—C9—C10	−0.5 (3)	C21—C22—C23—C25	0.3 (3)
N2—C8—C9—C10	178.3 (2)	N5—C22—C23—C24	1.7 (3)
C7—N1—C10—C9	0.1 (3)	C21—C22—C23—C24	−178.7 (2)
C7—N1—C10—C11	179.6 (2)	C25—C23—C24—F11	−116.5 (3)
C8—C9—C10—N1	−0.3 (4)	C22—C23—C24—F11	62.5 (3)
C8—C9—C10—C11	−179.8 (2)	C25—C23—C24—F10	122.1 (3)
N1—C10—C11—F4B	−18.2 (8)	C22—C23—C24—F10	−59.0 (3)
C9—C10—C11—F4B	161.4 (8)	C25—C23—C24—F12	3.8 (4)
N1—C10—C11—F5A	90.1 (11)	C22—C23—C24—F12	−177.3 (2)
C9—C10—C11—F5A	−90.3 (11)	C22—C23—C25—C26	1.2 (4)
N1—C10—C11—F6	−146.1 (2)	C24—C23—C25—C26	−179.8 (3)
C9—C10—C11—F6	33.5 (3)	C23—C25—C26—C27	−0.9 (4)
N1—C10—C11—F5B	115.6 (12)	C25—C26—C27—C21	−1.0 (4)
C9—C10—C11—F5B	−64.9 (12)	C22—C21—C27—C26	2.4 (4)
N1—C10—C11—F4A	−45.7 (13)	C20—C21—C27—C26	−177.9 (2)
C9—C10—C11—F4A	133.9 (13)	N7—N6—C28—C29	1.3 (3)
N3—N2—C12—C13	0.0 (3)	C20—N6—C28—C29	−179.8 (2)
C8—N2—C12—C13	174.1 (2)	N7—N6—C28—C32	−173.9 (2)
N3—N2—C12—C16	179.8 (2)	C20—N6—C28—C32	4.9 (4)
C8—N2—C12—C16	−6.0 (4)	N7—N8—C29—C28	1.3 (3)
N3—N4—C13—C12	−0.3 (3)	N7—N8—C29—C30	−179.3 (2)
N3—N4—C13—C14	178.4 (2)	N6—C28—C29—N8	−1.6 (3)
N2—C12—C13—N4	0.2 (3)	C32—C28—C29—N8	173.0 (3)
C16—C12—C13—N4	−179.7 (3)	N6—C28—C29—C30	179.1 (2)
N2—C12—C13—C14	−178.4 (3)	C32—C28—C29—C30	−6.3 (5)
C16—C12—C13—C14	1.8 (5)	N8—C29—C30—O2	170.1 (3)
N4—C13—C14—O1A	−175 (3)	C28—C29—C30—O2	−10.6 (4)
C12—C13—C14—O1A	4(3)	N8—C29—C30—C31	−10.1 (4)
N4—C13—C14—O1B	163 (3)	C28—C29—C30—C31	169.2 (3)

### Hydrogen-bond geometry (Å, °)

**Table:**

*D*—H···*A*	*D*—H	H···*A*	*D*···*A*	*D*—H···*A*
C3—H3···F3	0.93	2.33	2.677 (3)	102
C5—H5···F12^i^	0.93	2.51	3.178 (3)	129
C9—H9···N7^ii^	0.93	2.47	3.312 (4)	151
C16—H16A···O2^iii^	0.96	2.49	3.359 (4)	150
C25—H25···F12	0.93	2.31	2.659 (3)	102
C32—H32B···F5A^iv^	0.96	2.41	3.324 (14)	158

Symmetry codes: (i) ; (ii) *x*+1, *y*, *z*; (iii) *x*+1, *y*−1, *z*; (iv) *x*−1, *y*+1, *z*.
